# Temporal heterogeneity in photosystem II photochemistry in *Artemisia ordosica* under a fluctuating desert environment

**DOI:** 10.3389/fpls.2022.1057943

**Published:** 2022-11-03

**Authors:** Chuan Jin, Tianshan Zha, Charles P.-A. Bourque, Xin Jia, Yun Tian, Peng Liu, Xinhao Li, Xinyue Liu, Xiaonan Guo, Mingze Xu, Xiaoyu Kang, Zifan Guo, Ning Wang

**Affiliations:** ^1^ Yanchi Research Station, School of Soil and Water Conservation, Beijing Forestry University, Beijing, China; ^2^ Key Laboratory for Soil and Water Conservation, State Forestry and Grassland Administration, Beijing Forestry University, Beijing, China; ^3^ Faculty of Forestry and Environmental Management, University of New Brunswick, Fredericton, NB, Canada; ^4^ School of Land Science and Space Planning, Hebei GEO University, Shijiazhuang, China

**Keywords:** arid regions, chlorophyll fluorescence, desert plant, diurnal variation, seasonal fluctuations, wavelet analysis

## Abstract

Acclimation strategies in xerophytic plants to stressed environmental conditions vary with temporal scales. Our understanding of environmentally-induced variation in photosystem II (PSII) processes as a function of temporal scales is limited, as most studies have thus far been based on short-term, laboratory-controlled experiments. In a study of PSII processes, we acquired near-continuous, field-based measurements of PSII-energy partitioning in a dominant desert-shrub species, namely *Artemisia ordosica*, over a six-year period from 2012–2017. Continuous-wavelet transformation (CWT) and wavelet coherence analyses (WTC) were employed to examine the role of environmental variables in controlling the variation in the three main PSII-energy allocation pathways, i.e., photochemical efficiency and regulated and non-regulated thermal dissipation, i.e., *Φ*_PSII_, *Φ*_NPQ_, and *Φ*_NO_, respectively, across a time-frequency domain from hours to years. Convergent cross mapping (CCM) was subsequently used to isolate cause-and-effect interactions in PSII-energy partitioning response. The CWT method revealed that the three PSII-energy allocation pathways all had distinct daily periodicities, oscillating abruptly at intermediate timescales from days to weeks. On a diurnal scale, WTC revealed that all three pathways were influenced by photosynthetically active radiation (*PAR*), air temperature (*T*_a_), and vapor pressure deficit (*VPD*). By comparing associated time lags for the three forms of energy partitioning at diurnal scales, revealed that the sensitivity of response was more acutely influenced by *PAR*, declining thereafter with the other environmental variables, such that the order of influence was greatest for *T*_a_, followed by *VPD*, and then soil water content (*SWC)*. PSII-energy partitioning on a seasonal scale, in contrast, displayed greater variability among the different environmental variables, e.g., *Φ*_PSII_ and *Φ*_NO_ being more predisposed to changes in *T*_a_, and *Φ*_NPQ_ to changes in *VPD*. CCM confirmed the causal relationship between pairings of PSII-energy allocation pathways, according to shrub phenology. *A. ordosica* is shown to have an innate ability to (i) repair damaged PSII-photochemical apparatus (maximum quantum yield of PSII photochemistry, with *F*_v_/*F*_m_ > 0.78), and (ii) acclimatize to excessive *PAR*, dry-air conditions, and prolonged drought. *A. ordosica* is relatively sensitive to extreme temperature and exhibits photoinhibition.

## Introduction

Drylands, which make up almost half the earth’s continental area, have been expanding at an alarming rate as regional-to-global climate continues to deteriorate and human activity increases (Huang et al., 2017; Li et al., 2021). Consequently, plants in drylands (arid and semiarid lands) are frequently exposed to environmental stressors, triggered by excessive solar radiation, extreme temperature, drought, and other climatic anomalies (Jia et al., 2014; Tominaga et al., 2014). Consequently, understanding how dryland plants can cope with their harsh surroundings is of great importance to land managers and ecologists worldwide.

**Figure 1:**
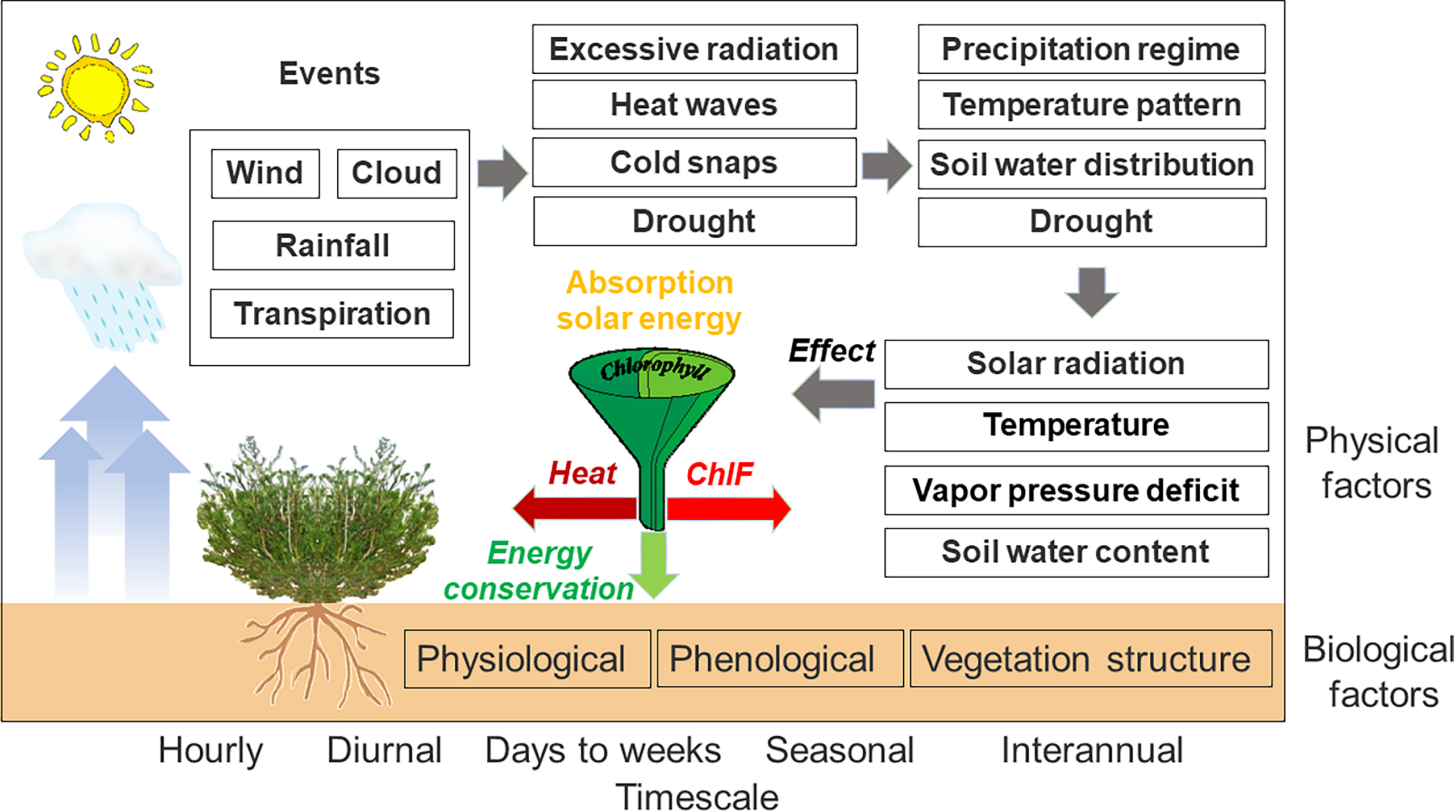
A conceptual description of biophysical factors that drive PSII-energy partitioning over multiple timescales. Drivers of PSII-energy partitioning included in this diagram are by no means comprehensive.

As a basis for maintaining energy and material flows in ecosystems, plant photosynthesis is particularly susceptible to environmental fluctuations, especially when representing extreme departures from favorable conditions (Schurr et al., 2006; Rodríguez-Calcerrada et al., 2008; Kalaji et al., 2012). Photosystem II (PSII) energy partitioning is considered the most sensitive element of photosynthesis (Quaas et al., 2015; Ni et al., 2019; Vilfan et al., 2019). Light energy collected by the light-trapping pigment in PSII is dissipated along three key energy-allocation pathways, i.e., (i) transfer to the photochemical reaction centers of photosynthesis, rated according to photochemical efficiency (*Φ*_PSII_), (ii) transfer as heat for thermal dissipation, and (iii) re-emission in the form of chlorophyll fluorescence (ChlF). Allocation along the three pathways is competitive. Heat avoidance in plants can occur by either regulatory or non-regulatory thermal dissipation, i.e., *Ф*_NPQ_ and *Ф*_NO_, respectively. Variables *Φ*_NPQ_ and *Φ*_NO_ represent the ability of photoprotection regulation and the extent of photoinhibition or photodamage in plants (Genty et al., 1989; Sperdouli and Moustakas, 2012). PSII-energy partitioning pathways, by way of *Φ*_PSII_, *Φ*_NPQ_, and *Φ*_NO_, are themselves affected by abiotic and biotic factors (**Figure 1**). These factors modulate the spectral characteristics of PSII-energy partitioning over multiple timescales, i.e., from seconds to minutes, to seasons (Stoy et al., 2009; Han et al., 2018; Jia et al., 2018). At sub-hour, minute timescales, for example, the intensification of photosynthetically active radiation (*PAR*) affects the xanthophyll cycle, causing excess light energy to be dissipated (Genty et al., 1989; Ruban et al., 2012; Ware et al., 2015). At hourly scales, in response to the increase in water vapor pressure (*VPD*), photochemical efficiency (*Ф*_PSII_) is lowered by stomatal closure (Nar et al., 2009; Zhou et al., 2013). At daily timescales, PSII-energy partitioning is largely driven by diel cycles of *PAR*, air temperature (*T*_a_), and *VPD* (Jia et al., 2014; Ouyang et al., 2014). At scales of several days to months, weather events accompanied by precipitation (*PPT*), high radiation levels, heat waves, cold spells influence photochemistry in PSII (Zha et al., 2017; Wu et al., 2018). At seasonal and interannual scales, PSII-energy partitioning may be affected by plant phenological processes and annual environmental biophysical cycles, particularly in soil water content (*SWC*; Han et al., 2018; Ren et al., 2018). Despite this past understanding, most studies on PSII-energy partitioning in plants have been based on short-term, laboratory-controlled experiments (Georgieva et al., 2005). However, under natural outdoor-conditions, PSII-energy partitioning response to fluctuations in local environmental conditions may be quite different over the short-to-long term (Sperdouli and Moustakas, 2012).

The pulse-amplitude modulation (PAM) technique facilitates the collection of near-continuous, *in situ* measurements of ChlF. Such measurements provide detailed information concerning the physiological state and performance of PSII in a rapid, non-destructive way (Ogawa et al., 2017; Stefanov et al., 2018). The method provides a direct assessment of the status of the three PSII-energy allocation pathways (Janka et al., 2015; Kalaji et al., 2017; Meacham et al., 2017). In general, interaction between fluctuating environmental and response variables is difficult to assess because of the presence of response delays (time lags) that may naturally differ across timescales. It is usually challenging to detect detailed information regarding time lags in covariances between PSII-energy partitioning and environmental variables by visual inspection of associated timeseries alone (Baldocchi et al., 2001; Zha et al., 2017). Although conventional methods of analysis, such as correlation analysis, can be used to quantify the contribution of environmental factors in controlling PSII-energy partitioning (Maseyk et al., 2019; Samson et al., 2019; Hikosaka, 2021; Sperdouli et al., 2021b), it is generally more difficult to untangle the multi-level interactions that naturally arise in complex systems. Spectral analysis, by means of wavelet analysis, for example, may yield valuable insights as to the temporal dynamics of PSII-energy partitioning and their biophysical forcing (Baldocchi et al., 2001; Qin et al., 2008; Ouyang et al., 2014). Compared to other spectral methods, wavelet analysis can exploit translation, expansion, and other functional operations in carrying out multi-scale analysis of several timeseries (Grinsted et al., 2004; Cazelles et al., 2008). Convergent cross mapping (CCM) is a nonparametric, statistical technique that helps to isolate cause-and-effect relationships in timeseries data (Sugihara et al., 2012; Chang et al., 2017). In contrast to simple linear correlation, CCM provides an improved explanation of nonlinear timeseries data, including detection of feedback, direction of causation, and linkages between dynamically-related variables (Chang et al., 2017). There are only a handful of scientific studies that apply both wavelet analysis and CCM to *in situ* ChlF-measured characteristics in desert-shrub ecosystems.

*Artemisia ordosica* is one of the most widespread shrub species in the Mu Us Desert of northwestern China. The species plays an important role in mitigating zones of quicksand and promoting community succession because of its deep rooting systems. The shrub species is particularly tolerant/resistant to being buried in sand for limited periods and to drought (Liu and Zhang, 2018). In this study, we applied continuous wavelet transform (CWT) and wavelet coherence (WTC) analyses on six years (2012–2017) of *in situ* measurements of ChlF and corresponding environmental variables (i.e., *PAR*, *T*_a_, *VPD*, and *SWC*) sourced at the same site. Convergent cross mapping was subsequently used to infer presence/absence of causality between pairings of the three PSII-energy partitioning pathways across timescales. The aim of the study was to (i) isolate the key controlling variables of PSII-energy partitioning in *A. ordosica* across multiple timescales, and (ii) evaluate the resilience of PSII in the shrub to harsh desert conditions. We hypothesized that across timescales, PSII-energy partitioning in *A. ordosica* is affected differently by the prevailing site-environmental conditions.

## Materials and methods

### Site characteristics

This study was carried out at the Yanchi Desert Ecosystem Research Station (37°53’83’’N, 107°25’46’’E; 1,530 m above mean sea level, amsl), Ningxia Hui Autonomous Region of northwestern China. The study area lies at the ecotone between the arid and semiarid zones of the desert, with *A. ordosica* (relative cover of 45%), *Salix psammophila* (20%), and *Hedysarum mongolicum* (20%) being the more abundant shrub species throughout the area. The prevailing climate is continental monsoon, where rainfall is rare and episodic. The mean annual precipitation (*PPT*) is 287 mm, mainly falling during the June–September period of each year. The mean annual potential evapotranspiration is about 2,024 mm, nearly an order of magnitude greater than *PPT*. The mean annual temperature is 8.3°C. All meteorological summaries are based on data from the Yanchi Meteorological Station (1954–2014), about 20 km from the research station.

### Long-term ChlF measurements and parameter calculation

Continuous ChlF measurements were acquired *in situ* during the growing seasons of 2012–2017 with a multi-channel PAM fluorometer (PAM 2000, Walz, Effeltrich, Germany). Five monitoring heads (MONI-head/485) connected to the fluorometer were installed on five different plants. A portion of healthy sun leaves were arranged in each MONI-head leaf clip. The sample branch was tied to an aluminum support inserted in the ground to ensure that the sample leaf clumps would not detach from the leaf clip or from the branch. The fluorometer used modulated blue LED light (450 nm wave peak and 18 nm bandwidth) to measure fluorescence emitted from the sample leaf clumps. The actinic light was based on natural sunlight. Positioning of the clipper heads were adjusted manually to avoid self-shading. The fluorometer measured fluorescence at the same frequency as modulated light (i.e., 5–25 Hz), so fluorescence could be measured across leaf-clump-physiological states, including during daytime conditions when sunlight was strongest. The two basic fluorescence parameters, namely real-time steady and maximal light-adapted fluorescence (*F*_t_ and *F*_m_’) were recorded automatically every 30 minutes with modulated light pulses set at 0.9 and 3500 µmol m^−2^ s^−1^, respectively. Since measurements of minimal and maximal dark-adapted fluorescence (*F*_0_ and *F*_m_) required absence of ambient light, we regarded nighttime *F*_t_ and *F*_m_’ as *F*_0_ and *F*_m_, respectively.

The maximum quantum yield of PSII photochemistry (*F*_v_/*F*_m_), photochemical efficiency (*Φ*_PSII_), and regulatory and non-regulatory thermal dissipation (*Ф*_NPQ_ and *Ф*_NO_, respectively) were calculated as follows:


(1) $$\frac{F_{v}}{F_{m}}=\frac{F_{m}-F_{0}}{F_{m}},$$

, (2) ΦPSII=Fm'−FtFm'

, and (3) ΦNPQ=FtFm'−FtFm

. (4) $$\Phi_{\text{NO}}=\frac{F_{\text{t}}}{F_{m}}$$

Raw ChlF-data were processed using the batch-file feature of the WinControl-3 software. Half-hourly values of *F*_m_’< 100 (non-dimensional) were considered atypical and removed from the dataset.

### Environmental measurements

Incident photosynthetically active radiation (*PAR*) and air temperature (*T*_a_) were measured simultaneously with the fluorometer. Relative humidity (*RH*) was measured with a thermohygrometer (HMP155A, Vaisala, Vantaa, Finland) mounted on a 6-m tall, eddy-covariance flux tower situated approximately 100 m from the ChlF-measurement area. Vapor pressure deficit (*VPD*) was derived from *T*_a_ and *RH* as discussed in Wilhelm et al. (1977), i.e.,


(5) $$VPD=0.611exp\;\;\left(\frac{17.27T_{\text{a}}}{T_{\text{a}}+237.3}\right)\times (1-\frac{RH}{100}).$$

Replicates of soil water content (*SWC*) were measured with moisture sensors placed at 30-cm depths (ECH_2_O-5TE, Decagon Devices, Pullman, WA, USA). *PPT* was quantified using a tipping-bucket raingauge installed 50 m from the ChlF-measurement area (TE525WS, Campbell Scientific Inc., Logan, UT, USA). All micrometeorological variables were averaged or summed every 30 minutes and stored on a datalogger.

### Data analysis

We subsequently analyzed the field data by means of continuous wavelet transform (CWT), cross-wavelet transform (XWT), wavelet coherence (WTC), and CCM (addressed below). Detailed reviews of wavelet analysis can be found in Grinsted et al. (2004) and Vargas et al. (2010). CWT was used to distinguish the timescales (hourly, daily, and so on) at which variability in timeseries (i.e., independent *vs.* dependent variables) is expressed. The CWT of a discrete timeseries (i.e., *x*_n_, with *n* = 1,…,N) recorded at a uniform timestep, *δ*_t_, is defined as the convolution integral of *x*_n_, with a scaled and normalized basis wavelet, *ψ*_0_(*η*). We write


, (6) $$W_{n}^{x}\left(s\right)=\sqrt{\frac{\delta_{t}}{s}}\sum_{n'=1}^{N}x_{n'}\psi_{0}^{*}\left[\frac{\left(n'-n\right)\delta_{t}}{s}\right]$$

where $\psi_{0}^{*}$ denotes the complex conjugate, and *s* is the set of wavelet scales applied (Cazelles et al., 2008). In this study, we chose a Morlet wavelet to serve as wavelet basis,


(7) $$\psi_{0}\left(\eta \right)=\;\pi^{-1/4}e^{iw_{0}\eta }e^{-\eta^{2}/2},$$

because it balances localization of frequency and time elapsed (Ouyang et al., 2014). From eqn. (6), we can define the wavelet power of *x*_n_ (i.e., *S*_n_) as


(8) $$S_{n}\left(s\right)={\left|W_{n}^{x}\left(s\right)\right|}^{2}.$$

Similarly, to quantify the spectral relationship between two timeseries, i.e., *x*_n_ (representative of *PAR*, *T*_a_, *VPD*, or *SWC*) and *y*_n_ (*Φ*_PSII_, *Φ*_NPQ_, and *Φ*_NO_), we defined the cross-wavelet power spectrum (*C*_n_), phase angle spectrum (*A*_n_), and WTC spectrum ( $R_{n}^{2}$ ), respectively, as


(9) $$C_{n}\left(s\right)=\left|W_{n}^{xy}\left(s\right)\right|=\left|W_{n}^{x}\left(s\right)W_{n}^{y*}\left(s\right)\right|,$$

, and (10) $$A_{n}\left(s\right)={\text{tan}}^{-1}\left[\frac{\text{Im}\left(W_{n}^{xy}\left(s\right)\right)}{\text{Re}\left(W_{n}^{xy}\left(s\right)\right)}\right]$$

, (11) $$R_{n}^{2}\left(s\right)=\frac{{\left|S\left(s^{-1}W_{n}^{xy}\left(s\right)\right)\right|}^{2}}{{\left|S\left(s^{-1}W_{n}^{x}\left(s\right)\right)\right|}^{2}{\left|S\left(s^{-1}W_{n}^{y}\left(s\right)\right)\right|}^{2}}$$

where *S* denotes a smoothing operation in both time and scale, which provides the minimal amount of smoothing necessary to include two independent points in both dimensions; $W_{n}^{xy}$ denotes XWT, and $\left[\text{Re}\left(W_{n}^{xy}\left(s\right)\right)\right]$ and $\left[\text{Im}\left(W_{n}^{xy}\left(s\right)\right)\right]$ are the real and imaginary parts of $W_{n}^{xy}\left(s\right)\;$ (Grinsted et al., 2004). The global cross-wavelet power spectrum (i.e., the mean of *C*_n_ over time) quantifies the magnitude of covariance that occurs between two timeseries across the frequency spectrum. Phase angles in *A*_n_ can indicate the time-frequency domain in *C*_n_ or $R_{n}^{2}$ . Arrows pointing right or left denote two timeseries that vary either in-phase or anti-phase. If two timeseries variables, i.e., *x*_n_ and *y*_n_, are positively correlated, phase arrows pointing upward indicate *x*_n_ lags *y*_n_ by a 1/4 period or leading *y*_n_ by a 3/4 period, while phase arrows pointing downward indicate *x*_n_ leads *y*_n_ by a 1/4 period or lags *y*_n_ by a 3/4 period. In this study, the interpretation of phase arrows related to the relationship between Φ_PSII_ and *PAR* should be seen as having an opposite response (i.e., arrows pointing left means that the variables are in-phase). All timeseries were normalized to have means of zero (with zeroes in data gaps) and unit variances. The statistical significance of wavelet spectra between two timeseries at a 5% critical significance level was evaluated within the cone of influence (COI), as per Vargas et al. (2010). Because of incomplete time-locality across frequencies, the wavelet transforms resulted in edge effects or artefacts (e.g., see below). Rather than use 10,000 Monte Carlo simulations as was done by the authors, we implemented 1,000 simulations. We performed wavelet analysis in MATLAB (R2018b, The MathWorks, USA) with codes acquired from Grinsted et al. (2004) and Ng and Chan (2012).

To complement the information provided by wavelet analysis, we subsequently used CCM to determine the direction and strength of causality between pairings of the three PSII-energy partitioning. The procedure is based on open-source scripts written in R. The scripts consisted of *multispatialCCM v*. 1.0 (Sugihara et al., 2012; Chang et al., 2017) and *pdc v.* 1.0.3 (via subroutine *entropyHeuristics*). Input requirements to run *multispatialCCM* were the timeseries embedded dimensions (E) and time delay (τ) determined with *pdc*. For hourly data, we randomly extracted 40 individual days from each phenological period over the six-year study period, as 960 consecutive records. Calculation of the standard deviation was based on 1,000 bootstrapping iterations.

## Results

### Seasonal dynamics of environmental variables and ChlF parameters

**Figure 2:**
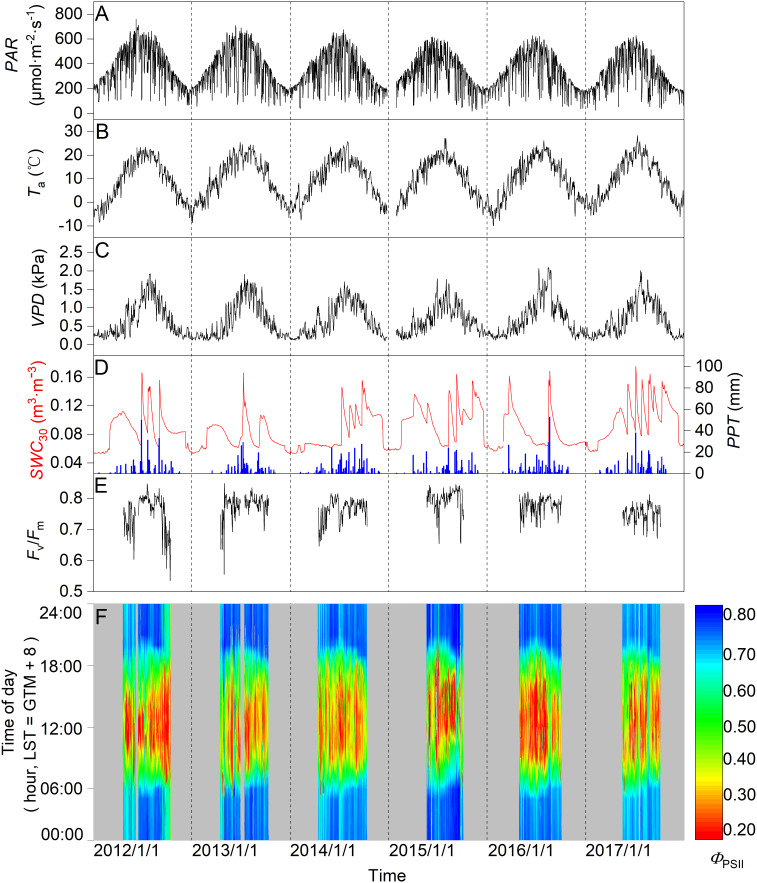
Seasonal variation in daily mean **(A)** photosynthetically active radiation (*PAR*), **(B)** air temperature (*T*_a_), **(C)** vapor pressure deficit (*VPD*), **(D)** soil water content at a 0.3-m depth (*SWC*), and daily **(D)** total precipitation (*PPT*; column), **(E)** maximal quantum yield of PSII photochemistry (*F*_v_/*F*_m_), and **(F)** photochemical efficiency (*Φ*_PSII_) for the 2012–2017 study period.

**Figure 2** reveals obvious seasonal patterns in *PAR*, *T*_a_, *VPD*, *SWC*, and *PPT* across the 2012–2017 study period. Daily mean *PAR* had a maximum of about 762 µmol m^−2^ s^−1^ in summer (**Figure 2A**), whereas daily mean *T*_a_ ranged from about -9.9°C in winter to 27.2°C in summer. Daily mean *VPD* varied from near-zero to 2.1 kPa in summer (**Figure 2C**). Seasonally, *SWC* exhibited clear pulse variations and wetting-to-drying cycles with a lower and upper limit of 0.05 to 0.17 m^3^ m^−3^, increasing abruptly in response to intermittent rainfall. Distribution of *PPT* was uneven, mainly concentrated in summer, ranging from 278.4–362.7 mm annually. *SWC* decreased in winter and sharply increased during the frost-thaw period in early spring (**Figure 2D**).

Seasonal changes in *F*_v_/*F*_m_ stabilized at around 0.77, with extremes (min-max) of 0.53 and 0.85, respectively (**Figure 2E**). Seasonally, the mode of diurnal *Φ*_PSII_ differed during the six-year period, varying between 0.10–0.88 (**Figure 2F**). Depressions in nighttime *F*_v_/*F*_m_ and *Φ*_PSII_ were observed across some years, e.g., 2012, 2013, and 2015, with mid-season drought when *SWC*< 0.10 m^3^ m^−3^ (**Figures 2D–F**).

### Diurnal variability in environmental variables and PSII-energy partitioning

**Figure 3:**
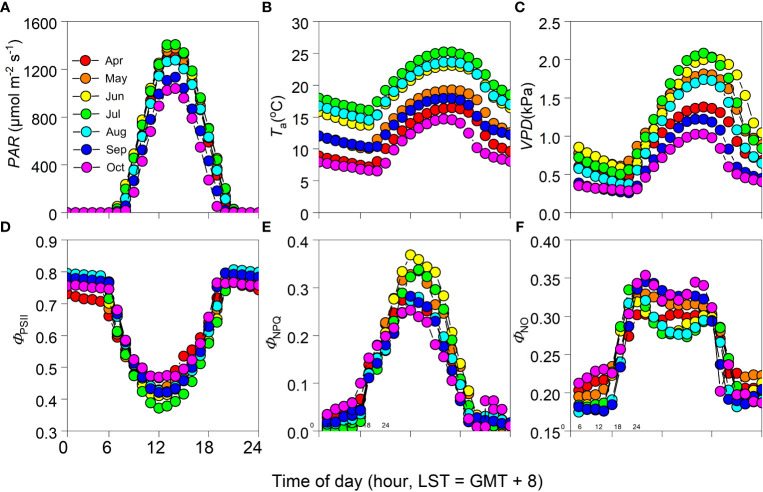
Monthly mean diurnal cycle of **(A)** photosynthetically active radiation (*PAR*), **(B)** air temperature (*T*_a_), **(C)** vapor pressure deficit (*VPD*), **(D)** photochemical efficiency (*Φ*_PSII_), and **(E)** regulatory and **(F)** non-regulatory thermal dissipation (*Φ*_NPQ_ and *Φ*_NO_, respectively) during the growing seasons of 2012–2017 (i.e., April–October period of each year).

Monthly mean variations in *PAR*, *T*_a_, and *VPD* had obvious diurnal patterns, peaking at about 13:00 [Beijing Standard Time (BST) = Greenwich Mean Time (GMT) + 8 hours] for *PAR* and 16:00 for both *T*_a_ and *VPD* (**Figures 3A–C**). During the June–August period of each year, *T*_a_ and *VPD* attained their highest values relative to the other times of the year (**Figures 3B, C**). Diurnally, *Φ*_PSII_ exhibited an opposite trend to that observed in *PAR*, producing a U-shaped curve, with its lowest value occurring at around 13:00 (**Figures 3A, D**). In contrast, temporal patterns in *Φ*_NPQ_ tended to match those observed in *PAR*, with its highest value occurring when *PAR* reached its maximum (**Figures 3A, E**). Diurnal patterns in *Φ*_NO_ were like those in *Φ*_NPQ_, having reached a plateau from 10:00–15:00 (**Figures 3E, F**). Daytime *Φ*_PSII_ and *Φ*_NO_ were generally lowest during the June–August period of the year, with *Φ*_NPQ_ reaching its highest value during that time (**Figures 3D–F**).

### Periodicity of environmental variables and PSII-energy partitioning

**Figure 4:**
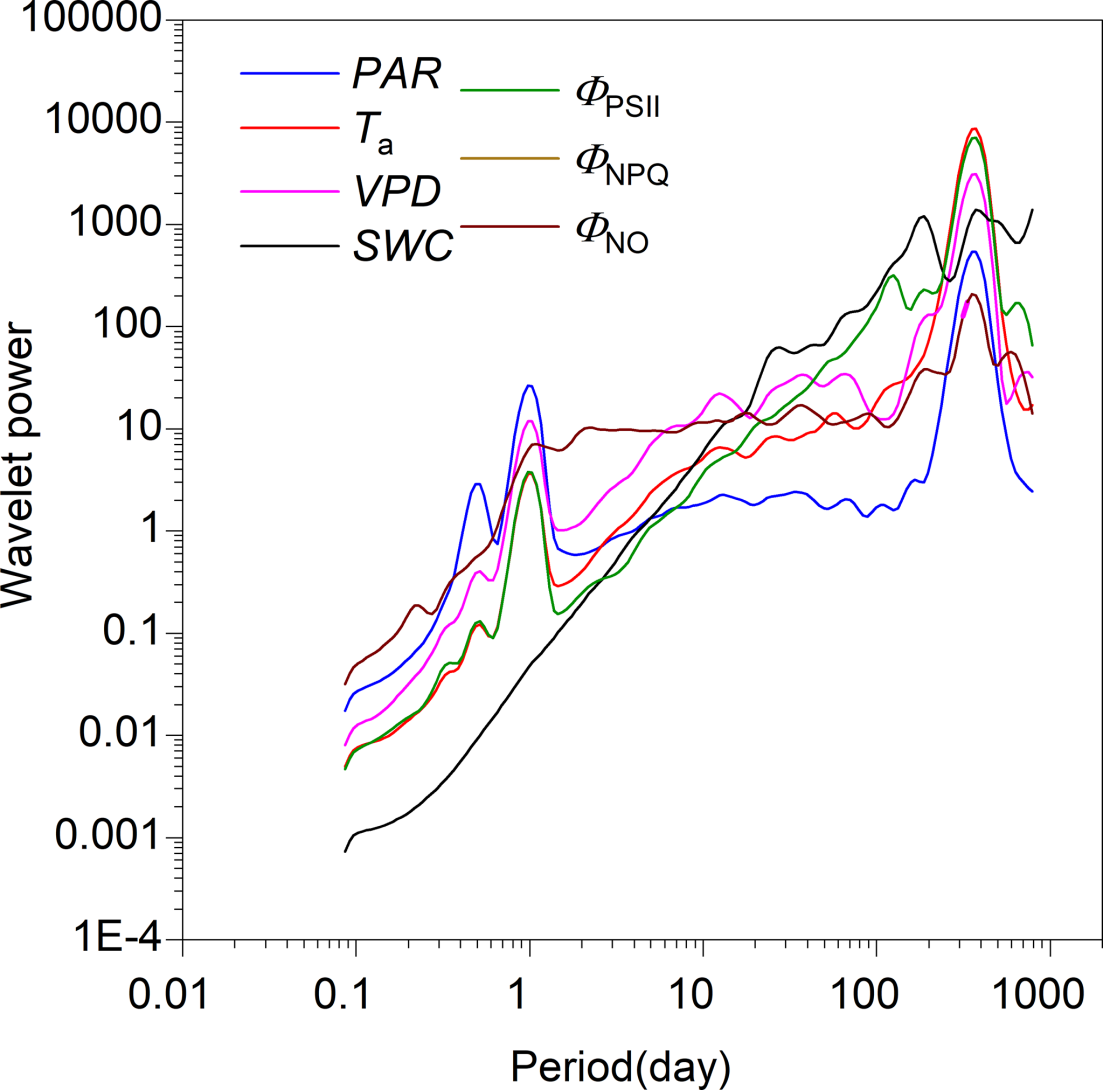
Global wavelet power spectra for photosynthetically active radiation (*PAR*), air temperature (*T*_a_), vapor pressure deficit (*VPD*), soil water content at a 0.3-m depth (*SWC*), photochemical efficiency (*Φ*_PSII_), and regulatory and non-regulatory thermal dissipation (*Φ*_NPQ_ and *Φ*_NO_, respectively).

According to the global wavelet power spectra, as expected, all timeseries showed timescale characteristics of periodicity, with a peak in power spectra corresponding with timescales of one day (except *SWC*) and 365 days (**Figure 4**). Meanwhile, *PAR*, *T*_a_, and *Φ*_PSII_ displayed a level of periodicity at sub-daily timescales, with associated power spectra being lowest. All timeseries oscillated strongly at intermediate timescales (i.e., days to weeks; **Figure 4**). Consistent with peaks in the global power spectra, CWT revealed partial characteristics across the time-frequency domain (**Figure S1**).

### Correlation at diurnal timescales

**Figure 5:**
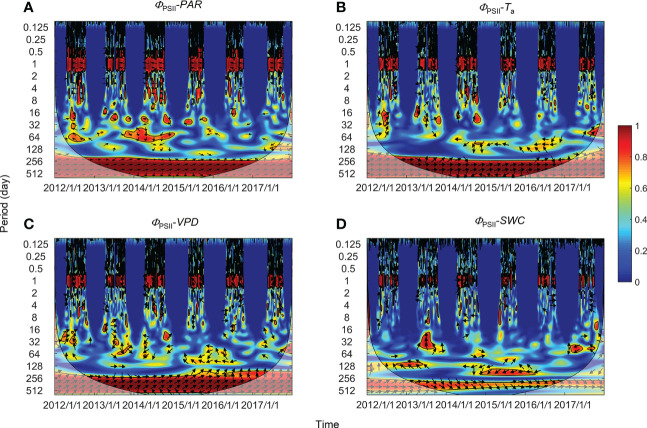
Wavelet coherence between photochemical efficiency (*Φ*_PSII_) and **(A)** photosynthetically active radiation (*PAR*), **(B)** air temperature (*T*_a_), **(C)** vapor pressure deficit (*VPD*), and **(D)** soil water content at a 0.3-m depth (*SWC*). The phase difference is shown by arrows. Arrows pointing upward indicate environmental factors leading *Φ*_PSII_ by 90°, whereas arrows pointing downward indicate environmental factors leading *Φ*_PSII_ by 270°. Arrows pointing left (or right) indicate environmental factors and *Φ*_PSII_ vary in-phase (or anti-phase). Black contour lines represent the 0.05 critical significance level. The thin arced lines denote the cone of influence (COI) that delimits the region not affected by edge artefacts.

**Figure 6:**
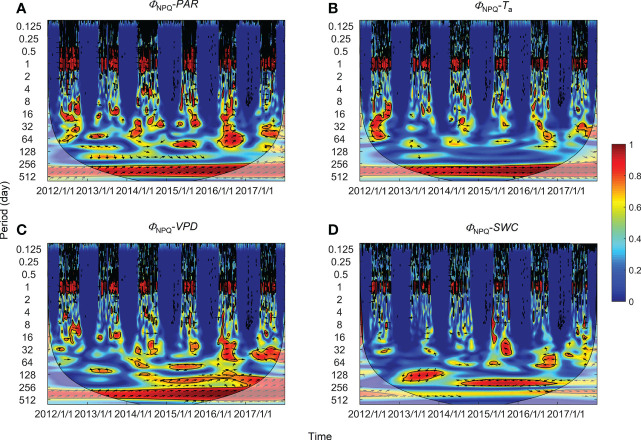
Wavelet coherence between non-regulatory thermal dissipation (*Φ*_NO_) and **(A)** photosynthetically active radiation (*PAR*), **(B)** air temperature (*T*_a_), **(C)** vapor pressure deficit (*VPD*), and **(D)** soil water content at a 0.3-m depth (*SWC*). The phase difference is shown by arrows. Arrows pointing upward indicate environmental factors leading *Φ*_NPQ_ by 90°, whereas arrows pointing downward indicate environmental factors leading *Φ*_NPQ_ by 270°. Arrows pointing left (or right) indicate environmental factors and *Φ*_NPQ_ vary in-phase (or anti-phase). Black contour lines represent the 0.05 critical significance level. The thin arced lines denote the cone of influence (COI) that delimits the region not affected by edge artefacts.

**Figure 7:**
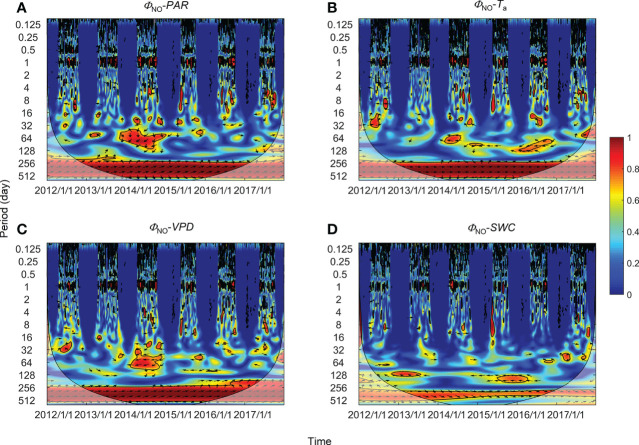
Wavelet coherence between regulatory thermal dissipation (*Φ*_NPQ_) and **(A)** photosynthetically active radiation (*PAR*), **(B)** air temperature (*T*_a_), **(C)** vapor pressure deficit (*VPD*), and **(D)** soil water content at a 0.3-m depth (*SWC*). The phase difference is shown by arrows. Arrows pointing upward indicate environmental factors leading *Φ*_NPQ_ by 90°, whereas arrows pointing downward indicate environmental factors leading *Φ*_NPQ_ by 270°. Arrows pointing left (or right) indicate environmental factors and *Φ*_NPQ_ vary in-phase (or anti-phase). Black contour lines represent the 0.05 critical significance level. The thin arced lines denote the cone of influence (COI) that delimits the region not affected by edge artefacts.

**Figure 8:**
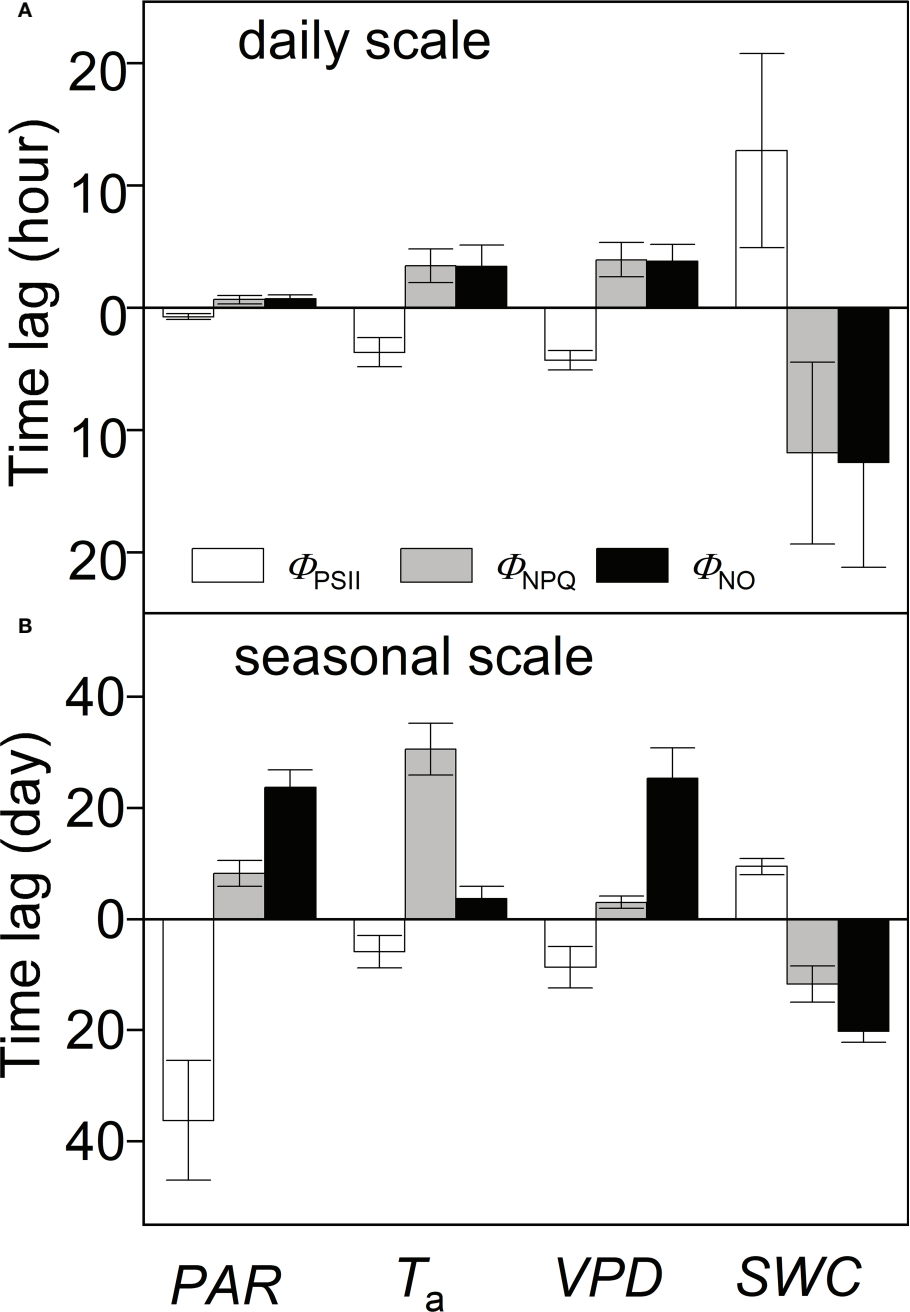
Time lags between PSII-energy partitioning measures and environmental factors at **(A)** daily and **(B)** seasonal timescales; *Φ*_PSII_ = photochemical efficiency; *Φ*_NPQ_ = regulatory thermal dissipation; *Φ*_NO_ = non-regulatory thermal dissipation; *PAR* = photosynthetically active radiation; *T*_a_ = air temperature; *VPD* = vapor pressure deficit; and *SWC* = soil water content at a 0.3-m depth. Columns above the central line (at time lag = zero) indicate positive correlation, whereas columns below the line indicate negative correlation. Data represent mean ± standard deviation (error bars).

Significant wavelet coherence (i.e., WTC) was observed between PSII-energy partitioning and environmental factors at the diurnal scale throughout the growing seasons (**Figures 5**–**7**). Diurnal variations in *Φ*_PSII_ were anti-phase with variations in *PAR* (i.e., arrows pointing left; **Figure 5A**). For example, the phase angle between *Φ*_PSII_ and *PAR* was -11.28 ± 3.43° (mean ± standard deviation), with *Φ*_PSII_ lagging *PAR* by 0.75 ± 0.23 h (**Figure 5A**; **Table 1**). For the same environmental variables, the time lags for the three PSII-energy partitioning pathways were roughly the same. The time lags in *PAR* were the shortest, about 40 minutes, followed by those of *T*_a_ and *VPD* at about 3.5 and 4 hours, respectively. The time lags in *SWC* were the longest at about 12 hours (**Figure 8A**).

**Table 1:** Time lags between PSII-energy partitioning along the three pathways and environmental factors on a daily timescale [i.e., photosynthetically active radiation (*PAR*), air temperature (*T_a_*), vapor pressure deficit (*VPD*), and soil water content at a 0.3-m depth (*SWC*)].

PSII-energy partitioningpathways	*PAR*	*T*_a_	*VPD*	*SWC*
Photochemical efficiency (*Φ*_PSII_)	0.75 ± 0.23	3.66 ± 1.21	4.32 ± 0.80	12.83 ± 7.93
Regulatory thermal dissipation (*Φ*_NPQ_)	0.64 ± 0.33	3.40 ± 1.37	3.90 ± 1.41	11.90 ± 7.42
Non-regulatory thermal dissipation (*Φ*_NO_)	0.72 ± 0.30	3.34 ± 1.77	3.79 ± 1.38	12.65 ± 8.61

Note, that the values in the Table are the leading times and associated standard deviations. The unit of time is hours.

### Correlation at intermediate and annual timescales

High global wavelet power was found between the PSII-energy partitioning pathways and environmental factors at periods between 10–100 days (see **Figure S1**). Intermittent areas of statistically significant WTC were observed at timescales between 16–128 days throughout the growing seasons (**Figures 5**–**7**). For instance, bands and hotspots in WTC were found in pairings of *Φ*_PSII_-to-*PAR*, *Φ*_NO_-to-*PAR*, and *Φ*_NO_-to-*VPD* at about 64 days over the 2015 growing season (**Figures 5**, **6**). Both *PAR* and *VPD* showed strong WTC with their pairings with *Φ*_NPQ_ at 64–128-day intervals during the 2016 growing season (**Figure 7**).

In comparison with diurnal timescales, the time lags between individual PSII-energy partitioning and environmental variables showed some level of discrepancy at intermediate timescales (**Table 2**). For *PAR*, time lag in *Φ*_NPQ_ was significantly shorter than the time lags for both *Φ*_NPQ_ and *Φ*_NO_ (i.e., 8.23 *vs.* > 20 days), whereas for *T*_a_, the time lags in *Φ*_NO_ and *Φ*_PSII_ were shorter than the time lag in *Φ*_NPQ_, i.e., 3.72 and 5.89 days, respectively, *vs.* 30.51 days. Again, in terms of *VPD*, the time lags in *Φ*_NPQ_ and *Φ*_PSII_ were shorter than the time lag in *Φ*_NO_, giving 3.01 and 8.67 days, respectively, *vs.* 25.29 days. The time lags for *SWC* were moderate, ranging between 9.43 and 20.18 days (**Figure 8B**).

**Table 2:** Time lags between PSII-energy partitioning pathways and environmental factors on a monthly timescale [i.e., photosynthetically active radiation (*PAR*), air temperature (*T_a_*), vapor pressure deficit (*VPD*), and soil water content at a 0.3-m depth (*SWC*)].

PSII-energy partitioningpathways	*PAR*	*T*_a_	*VPD*	*SWC*
Photochemical efficiency (*Φ*_PSII_)	36.21 ± 10.79	5.89 ± 2.92	8.67 ± 3.68	9.43 ± 1.49
Regulatory thermal dissipation (*Φ*_NPQ_)	8.23 ± 2.33	30.51 ± 4.65	3.01 ± 1.11	11.71 ± 3.23
Non-regulatory thermal dissipation (*Φ*_NO_)	23.65 ± 3.19	3.72 ± 2.12	25.29 ± 5.49	20.18 ± 1.98

Note, that the values in the Table are the leading times and associated standard deviations. The unit of time is days.

### Causal inference at multiple timescales

Appearing in **Table 3** are the results of CCM based on hourly PSII-energy partitioning as a function of shrub phenology. At the hour scale, causality among pairings of energy allocation measures was mostly in agreement across the various phenological periods. Parameter *Φ*_PSII_ had a unidirectional causal relationship with *Φ*_NPQ_, with *Φ*_PSII_ forcing *Φ*_NPQ_ (case II). Except when *Φ*_PSII_-forcing-*Φ*_NO_ was not statistically significant (*p* > 0.05) during the budding period, both *Φ*_NPQ_ and *Φ*_PSII_ had a bidirectional causal connection (feedback) with *Φ*_NO_ (case III). In contrast, causal relationships among daily PSII-energy partitioning measures varied across phenological phases (**Table 4**); *Φ*_PSII_ was shown not to be causally related to *Φ*_NPQ_ (case I), whereas *Φ*_NPQ_ was bidirectionally related to *Φ*_NO_ (case III). The unidirectional causality between *Φ*_PSII_ and *Φ*_NO_ (case II) changed from *Φ*_PSII_ forcing *Φ*_NO_ during the budding and leaf-expanded phases to *Φ*_NO_ forcing *Φ*_PSII_ during the leaf-coloring phase.

**Table 3:** Convergent cross mapping (CCM) with hourly data pairs relating three PSII-energy partitioning pathways for different phenological phases from 2012–2017 (*L*_n_ ~ 960 records); the number of bootstrapping iterations (n) = 1,000; and A|B and B|A stand for A forcing B and B forcing A, respectively.

Variable A, B	Phenological phase	*p*-value A|B,B|A	ρ_∞_ A|B,B|A	r^2^ A|B,B|A	Causality
*Φ*_PSII_, *Φ*_NPQ_	budding	0.01**	0.43	0.94	II
		0.64	–	–	
	leaf-expanded	0.01**	0.42	0.87	II
		0.53	–		
	leaf-coloring	0.01**	0.49	0.92	II
		0.23	–	–	
*Φ*_NPQ_, *Φ*_NO_	budding	0.01**	0.77	0.96	III
		0.03*	0.54	0.94	
	leaf-expanded	0.01**	0.57	0.99	III
		0.01**	0.62	0.90	
	leaf-coloring	0.01**	0.61	0.98	III
		0.01**	0.68	0.98	
*Φ*_PSII_, *Φ*_NO_	budding	0.08	–	–	II
		0.01**	0.94	0.98	
	leaf-expanded	0.01**	0.88	0.93	III
		0.01**	0.92	0.98	
	leaf-coloring	0.01**	0.78	0.83	III
		0.01**	0.79	0.91	

** and * indicate critical statistical significance at p-values< 0.01 and 0.05, respectively. The Roman numerals in the sixth column coincide with the absence (case I) or presence of unidirectional causality (i.e., A|B or B|A, designated as case II) or presence of bidirectional causality (i.e., A|B and B|A, designated as case III); r^2^ is the coefficient of determination associated with fitting the Michaelis-Menten equation [i.e., y = *a*x/(*b* + x)]; ρ_∞_ (or parameter *a* in the equation) gives the asymptote when *L* → *L*_n_.

**Table 4:** Convergent cross mapping (CCM) with daily data pairs relating three PSII-energy partitioning pathways for different phenological phases from 2012–2017 (*L*_n_ ~ 202, 528, and 230 records); the number of bootstrapping iterations (n) = 1,000; and A|B and B|A stand for A forcing B and B forcing A, respectively.

Variable A, B	Phenological phase	*p*-value A|B,B|A	ρ_∞_ A|B,B|A	r^2^ A|B,B|A	Causality
*Φ*_PSII_, *Φ*_NPQ_	budding	0.24	–	–	I
		0.44	–	–	
	leaf-expanded	0.30	–	–	I
		0.33	–	–	
	leaf-coloring	0.40	–	–	I
		0.18	–	–	
*Φ*_NPQ_, *Φ*_NO_	budding	0.01**	0.60	0.99	III
		0.01**	0.60	0.98	
	leaf-expanded	0.01**	0.85	0.99	III
		0.01**	0.80	0.99	
	leaf-coloring	0.03*	0.71	0.91	III
		0.01**	0.75	0.98	
*Φ*_PSII_, *Φ*_NO_	budding	0.04*	0.33	0.83	II
		0.56	–	–	
	leaf-expanded	0.03*	0.32	0.96	II
		0.19	–	–	
	leaf-coloring	0.27	–	–	II
		0.02*	0.47	0.85	

** and * indicate critical statistical significance at p-values< 0.01 and 0.05, respectively. The Roman numerals in the sixth column coincide with the absence (case I) or presence of unidirectional causality (i.e., A|B or B|A, designated as case II) or presence of bidirectional causality (i.e., A|B and B|A, designated as case III); r^2^ is the coefficient of determination associated with fitting the Michaelis-Menten equation [i.e., y = *a*x/(*b* + x)]; ρ_∞_ (or parameter *a* in the equation) gives the asymptote when *L* → *L*_n_.

## Discussion

### Environmental controls at diurnal timescales

The fate of excited states formed in PSII is variable and depends on the physiological history and state of the leaves. Events of extreme temperatures (Yan et al., 2011), drying (Ma et al., 2015), high rates of transpiration, and excessive solar radiation (Dai et al., 1992; Han et al., 2018), all have the potential to accelerate photoinhibition or photodamage in plants by affecting the energy absorbed and partitioned in PSII (Suzuki et al., 2014; Sacharz et al., 2017). Correspondingly, plants can dissipate a major part of this surplus light energy to offset the impact of reactive-oxygen-species accumulation to avoid photodamage (Wilhelm and Selmar, 2011; Zhang et al., 2021).

WTC-results suggested that responses (time lags) to variations in *PAR* were fastest among the PSII-energy partitioning pathways (**Figure 8A**). Solar radiation in the summer largely exceeded the photosynthetic needs of plants. Here, the photochemical process and the heat dissipation were modulated almost immediately (Jahns and Holzwarth, 2012). Even though most of the excessive excitation energy can be safely dissipated as thermal energy, an overproduction of reactive-oxygen-species (ROS) is still inevitable, which can lead to photodamage (Chen et al., 2016; Yamori, 2016; Sperdouli et al., 2021a). In this study, *Φ*_PSII_ measured at night was equated as a reasonable substitute for *F*_v_/*F*_m_. Nonetheless, after the photochemical components recovered overnight, the measured expression of *F*_v_/*F*_m_, to some extent, was equivalent to the resilience in PSII (Zha et al., 2017). Variable *Φ*_PSII_ reached its lowest value at about 12:00 local time and was restored to its original value at night (**Figure 3D**), indicating that photochemical reactions in *A. ordosica* were limited by high levels of solar radiation, but its PSII-reaction centers avoided photodamage (Zhang et al., 2021). However, studies on the same subject found that *S. psammophila* underwent severe photoinhibition due to disproportionate levels of solar radiation, resulting in irreversible leaf damage (Han et al., 2018).

The average time lags for *T*_a_ and *VPD* with PSII-energy partitioning in *A. ordosica* were 3.5 and 4.0 h, respectively (**Table 1**). Some studies have confirmed that cold temperatures can inhibit the activity of chloroplast protein import, thus impacting the photosynthetic state of PSII (Leheny and Theg, 1994; Savitch et al., 2009). During early morning, with the lowest *T_a_* (*PAR* being close to zero), *Φ*_PSII_ showed a slight decline (**Figures 3B, D**), indicating that low temperatures may inhibit PSII in *A. ordosica.* The study found that extreme daytime temperatures served as a direct threat to PSII by affecting the activity of antioxidants and Calvin-Benson cycle enzymes (Liu et al., 2012). Unfortunately, we do not have direct evidence to specify exactly how extreme temperatures affected PSII in *A. ordosica*. Dry and wet air circulation (causing fluctuations in *VPD*) can induce changes in stomatal aperture. Closure of stomates can limit photosynthetic electron transfer and potentially activate photoinhibition (Ghimire et al., 2018; Bambach et al., 2020). Meanwhile, inhibited water transport can increase the proton-gradient across thylakoid membranes, and eventually cause *Φ*_PSII_ to decrease and *Φ*_NPQ_ and *Φ*_NO_ to increase (Johnson and Ruban, 2014).

Time lags in plant response to *SWC* were much longer than for the other environmental factors (**Figure 8A**), suggesting that *A. ordosica* is less sensitive to *SWC* during the short term (Wu et al., 2018). Overall, by comparing time lags, we propose that the sensitivity of PSII in *A. ordosica* at diurnal timescales is largely associated with variations in *PAR*, *T*_a_, and then *VPD* (**Table 1**). This suggests that diurnal heat dissipation and the change in stomatal conductance during the day are the main processes that regulate PSII-energy partitioning in *A. ordosica* at daily timescales.

### Environmental controls at intermediate scales

At seasonal timescales, harsh dryland environments are mainly characterized by excessive solar radiation and extreme temperatures lasting for several days to weeks, causing hydrological gradients to shift (Niinemets, 2010; Suzuki et al., 2014). Studies have shown that long-term adaptation mechanisms in PSII takes several days or even weeks to respond, which is achieved by adjusting photosynthetic pigment concentration and chlorophyll a-to-chlorophyll b ratios, or PSII-specific protein content (Müller et al., 2001; Savitch et al., 2009; Ren et al., 2018).

Our results show that time lags associated with each PSII-energy allocation pathway vary at seasonal timescales (**Figure 8B**). Short time lags of *Φ*_NPQ_-to-*PAR* pairings (8.23 days) indicate that *A. ordosica* has a regulatory PSII mechanism to respond to high levels of solar radiation. This is achieved by the shrub’s ability to actively increase rates of thermal dissipation (Wu et al., 2018; Samson et al., 2019). Excessive solar radiation breaks the stability between PSII-energy input and utilization, causing damage to the light-harvesting protein complexes (Stefanov et al., 2018). Time lags for the *Φ*_PSII_-to-*PAR* and *Φ*_NO_-to-*PAR* pairings can be up to 36.21 and 23.65 days, respectively (**Table 2**). This suggests that *A. ordosica* has a level of tolerance to surplus solar radiation. Morphologically, their needle-shaped leaves help to minimize excessive light capture (Liu and Zhang, 2018).

The seasonal temperature range in deserts is significant (**Figure 2B**). The short time lags associated with the *Φ*_PSII_-to-*T*_a_ and *Φ*_NO_-to-*T*_a_ pairings (i.e., 5.89 and 3.72 days, respectively) indicate that seasonal fluctuations in temperature threaten the stability of PSII, which can result in a decrease in photosynthetic capacity (Mathur et al., 2014). It is worth noting that *Φ*_NPQ_ lagged *T*_a_ by 30.51 days (**Table 2**), which can be viewed as a rather slow adjustment. It has been reported that desert plants can adapt to extreme temperatures by increasing the osmotic pressure in cells, increasing the anti-coagulant properties of protoplasm and reducing their metabolic rate (Faik et al., 2016; Wu et al., 2018).

Our results show that *VPD* impacts *Φ*_NPQ_ and *Φ*_PSII_ rapidly (about 3–8 days), whereas it influences *Φ*_NO_ much more slowly (~25 days). We conclude that under dry-air conditions, *A. ordosica* can tolerate the on-going conditions with their fully evolved stomatal structures and gelatinous mesophyll (Dai et al., 1992; Ghimire et al., 2018).

Under large hydraulic gradients, inactivation of PSII due to aridity stresses often alternates, causing reversible photodamage (Biederman et al., 2017; Wu et al., 2018). WTC-results show that during early drought (within ~10 days of its onset), *A. ordosica* can maintain a stable PSII with long-term regulatory capacity (Ghimire et al., 2018). Due to *A. ordosica*’s well-developed rooting system, with a thick taproot and dense lateral roots, uptake of soil water is maintained even under conditions of extended drought (Fan et al., 2017). Moreover, *F*_v_/*F*_m_ fluctuates with long-term, low growing-season *SWC*, maintaining an average *F*_v_/*F*_m_-ratio of 0.78 (**Figure 2E**). Our study submits that *A. ordosica* can recover through PSII self-repair and regulation under long-term drought.

### PSII-energy partitioning and its relationship to shrub phenology

One-way interactions between hourly *Φ*_PSII_ and *Φ*_NPQ_ identified with CCM, suggests that photosynthetic limitation in *A. ordosica* caused the shrub to mitigate pressure of excess light energy by regulating heat dissipation (**Table 3**). By contrast, the two-way interaction between hourly *Φ*_PSII_ and *Φ*_NO_ shows that inhibition of photosynthesis can result in photodamage. This photodamage, in turn, is expected to result in a simultaneous reduction in photosynthesis as a result of the variables’ mutual relationship in a negative feedback loop (Genty et al., 1989; Jahns and Holzwarth, 2012).

We also found that PSII-energy partitioning in *A. ordosica* was affected by its phenology. During budding, from April to early May of each year, daytime *Φ*_NO_ was shown to increase (**Figure 3F**). Low chlorophyll content and photosynthesis activity during this phase promoted photoinhibition (Ren et al., 2018). During the leaf-expanded phase (June–August of each year), *Φ*_NPQ_ was seen to increase and *Φ*_PSII_ to decrease (**Figures 3D, E**). During this period, biomass and leaf functional traits had reached their optimum functioning. Bidirectional causal relationships identified with daily *Φ*_NPQ_ and *Φ*_NO_ (**Table 4**), suggested that PSII in *A. ordosica* was most likely in a cycle of constant injury and mending (Han et al., 2018). During the leaf-coloring phase (i.e., September–October), daytime *Φ*_NO_ and *F*_v_/*F*_m_ were seen to increase and decrease, respectively (**Figures 2E**, **3F**), and the unidirectional causal relationship, i.e., *Φ*_PSII_ forcing *Φ*_NO_, changed to *Φ*_NO_ forcing *Φ*_PSII_ (**Table 4**). During this period, shrub chlorophyll content and enzyme activity rapidly decreased, resulting in further cumulative photodamage, increasing PSII-photochemical suppression.

## Conclusions

Functional dependencies between PSII-energy partitioning and environmental variables varied across timescales. These discrepancies reflect the adaptive strategies in *A. ordosica* to changing desert environments. Diurnally, all PSII-energy partitioning pathways were largely dominated by *PAR*, regulated to some extent by T_a_ and *VPD*, and exhibited low sensitivity to *SWC*. Seasonally, the PSII-energy partitioning in *A. ordosica* was affected by both physiological and phenological factors. Both *Φ*_PSII_ and *Φ*_NO_ were vulnerable to *T*_a_, whereas *Φ*_NPQ_ was most sensitive to *VPD*. Short-term drought (within< 10 days) had little effect on PSII-energy partitioning. *A. ordosica* was shown to have an ability to repair itself, with an *F*_v_/*F*_m_ > 0.78. Our results suggested that *A. ordosica* can acclimate to excessive *PAR*, air aridity, and prolonged drought, exhibiting rapid response to variation in extreme temperatures by means of photoinhibition. Our findings have important implications for understanding the adaptation capacity in dryland shrub species in desert-plant communities under current climate change. Information from this study is beneficial to combatting desertification and restoring ecological function to drylands globally.

## Data availability statement

The original contributions presented in the study are included in the article/**Supplementary Material**. Further inquiries can be directed to the corresponding author.

## Author contributions

TZ and CJ conceived the study. CJ, ZG, MX and NW conducted the fieldwork. CJ, XG, XK and XHL analyzed the data. CJ wrote the manuscript with the assistance of TZ and PL. CP-AB helped revise/polish the manuscript. YT and XJ revised the manuscript. All authors contributed to and approved the final manuscript.

## Funding

The research was supported by grants from the National Natural Science Foundation of China (NSFC: 32071842, 32101588, 31901366, 32071843), by the National Key Research and Development Program of China (No. 2020YFA0608100).

## Acknowledgments

We thank TZ Wei, WJ Zhou, and RZ Yang for their assistance with field measurements and instrument maintenance. The U.S.-China Carbon Consortium (USCCC) supported this work by way of helpful discussion and the exchange of scientific ideas.

## Conflict of interest

The authors declare that the research was conducted in the absence of any commercial or financial relationships that could be construed as a potential conflict of interest.

## Publisher’s note

All claims expressed in this article are solely those of the authors and do not necessarily represent those of their affiliated organizations, or those of the publisher, the editors and the reviewers. Any product that may be evaluated in this article, or claim that may be made by its manufacturer, is not guaranteed or endorsed by the publisher.

## Supplementary material

Supplementary Figure 1 Wavelet power spectra for daily mean **(A)** photosynthetically active radiation (*PAR*), **(B)** air temperature (*T*_a_), **(C)** vapor pressure deficit (*VPD*), **(D)** soil water content at a 0.3-m depth (*SWC*), **(E)** photochemical efficiency (*Φ*_PSII_), and **(F)** ratio of regulatory to non-regulatory thermal dissipation (*Φ*_NPQ_ and *Φ*_NO_, respectively) for the 2012–2017 study period. Black contour lines represent the 0.05 critical significance level. The thin arced lines denote the cone of influence (COI) that delimits the region not affected by edge artefacts. The color ranges from dark blue to dark red, coinciding with wavelet power spectra from low to high.
