# Supplementary figures and images for: Temporal heterogeneity in photosystem II photochemistry in Artemisia ordosica under a fluctuating desert environment
Source: Front Plant Sci. 2022 Nov 3;13:1057943. doi: 10.3389/fpls.2022.1057943 (PMC9670136; doi:10.3389/fpls.2022.1057943)

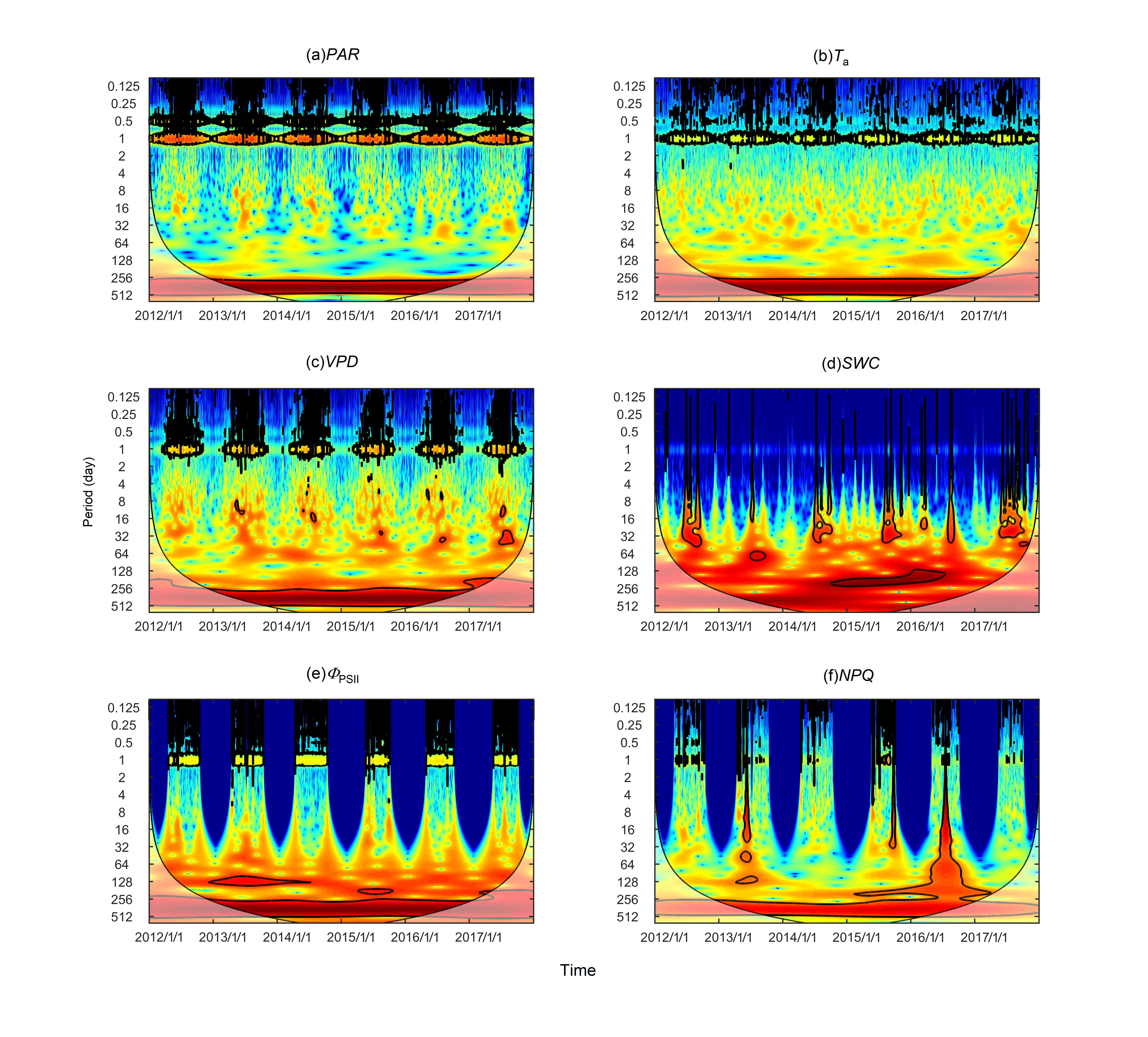

Supplement: Supplementary Figure 1 — Wavelet power spectra for daily mean (A) photosynthetically active radiation (PAR), (B) air temperature (T a), (C) vapor pressure deficit (VPD), (D) soil water content at a 0.3-m depth (SWC), (E) photochemical efficiency (Φ PSII), and (F) ratio of regulatory to non-regulatory thermal dissipation (Φ NPQ and Φ NO, respectively) for the 2012–2017 study period. Black contour lines represent the 0.05 critical significance level. The thin arced lines denote the cone of influence (COI) that delimits the region not affected by edge artefacts. The color ranges from dark blue to dark red, coinciding with wavelet power spectra from low to high. [file Image_1.tif]
